# Design and Implementation of the STM32N6-Based Modular Embedded Edge AI Teaching Platform for Engineering Education and Competition Practices

**DOI:** 10.3390/s26144631

**Published:** 2026-07-21

**Authors:** Zixuan Wang, Liguo Liu, Ping Wang, Jinzhe Wu

**Affiliations:** 1 School of Engineering, Naval University of Engineering, Wuhan 430030, China; 2320232037@nue.edu.cn (Z.W.); 1309021015@nue.edu.cn (L.L.); 2 College of Electronic and Information Engineering, Shandong University of Science and Technology, Qingdao 266590, China; 202511050918@sdust.edu.cn

**Keywords:** embedded edge AI, STM32N6, educational platform, PEPoseNet

## Abstract

This paper presents a modular embedded edge-AI teaching platform built around the STM32N6 microcontroller, designed to meet demand for low-power, real-time, deployable edge intelligence in engineering education. The platform uses a heterogeneous architecture combining an ARM Cortex-M55 core with a dedicated Neural-ART NPU, enabling efficient on-device inference for both classroom projects and vision-based competition tasks. To improve stability across multi-peripheral setups, a multi-power-domain supply architecture combines switched-mode power supplies with low-noise LDO regulators, plus dual-input power switching, reverse-current and reverse-polarity protection, overcurrent limiting, and soft-start control. High-speed modular peripherals are integrated on board—MIPI-CSI camera input, RGB display output, high-speed NOR Flash, and SPI, I2C, UART, and TIMER expansion interfaces—with an 80-pin board-to-board connector for flexible extension. A four-layer PCB layout improves signal integrity for reliable high-speed operation. Deploying a custom lightweight vision algorithm (PEPoseNet), the prototype achieves an inference-only latency of 18.4 ms and 28.31 FPS/Watt energy efficiency within a sub-3 W power budget. These results confirm the platform’s reliability as an educational training platform for embedded edge-AI computing.

## 1. Introduction

With the exponential proliferation of Internet of Things (IoT) devices, Edge Artificial Intelligence (Edge AI) has emerged as a core architectural paradigm for next-generation intelligent systems. By deploying lightweight neural networks directly on terminal devices, Edge AI fundamentally mitigates the latency, bandwidth, and privacy bottlenecks inherent in cloud reliance, enabling real-time applications such as hazard identification and industrial defect detection [1,2]. Consequently, embedded Edge AI has become a focal point in engineering education and university-level technological competitions, where tasks demand an optimal balance between low power consumption and high inference accuracy [3,4,5]. In this context, lightweight models such as quantized YOLO algorithms have become the de facto standard for resource-constrained deployments [6].

However, since the theoretical foundations related to embedded edge AI computing are not emphasized in theoretical instruction and there is a lack of corresponding laboratory sessions, there remains a significant gap between students’ understanding and mastery of edge AI computing and the competencies required to participate in university-level technological competitions [7]. Moreover, learning embedded edge AI computing involves not only the development and utilization of microcontroller hardware resources but also considerable complexity in algorithms and software stacks [8]. Consequently, developing embedded edge AI systems requires interdisciplinary knowledge in areas such as digital circuits, database principles, embedded systems, and automatic control theory, placing high demands on students’ practical innovation and comprehensive application abilities [9,10].

Drawing on years of experience coaching students in edge-AI competitions, we identified a clear need for a dedicated innovation and practice platform for embedded edge-AI education. Such a platform would help students master core concepts while building hands-on skills, better preparing them for competition-level work.

Existing teaching platforms for competitions balance hardware performance with pedagogical adaptability, and their modular design principles inform this work [11]. Building on this foundation, our team analyzed and structured the embedded-AI requirements typical of university competitions, leading to the STM32N6-based teaching platform presented here.

Current literature and educational frameworks enforce a critical trade-off: educators must choose between computational capability (MPUs) and real-time, low-power control (MCUs). Very few studies have proposed a unified platform that bridges this gap. The platform proposed in this paper directly addresses this void by leveraging the heterogeneous architecture of the STM32N6 (Cortex-M55 + embedded 600-GOPS NPU; STMicroelectronics, Geneva, Switzerland). This architecture retains the real-time-oriented control characteristics of an MCU while providing NPU-accelerated edge inference under a strict power budget of less than 3 W. It therefore offers a practical hardware foundation for embedded edge-AI teaching and competition-oriented deployment. This platform is conceived and evaluated as an educational training prototype and open hardware teaching reference design, rather than as a commercial training kit or a general-purpose industrial vision system.

The novelty of the proposed work is not to invent a new microprocessor, a new AI toolchain, or a new neural-network framework. However, our contribution is the system-level educational co-design of hardware topology, edge-AI deployment workflow, and competition-oriented educational practice. Existing STM32 development board is designed from the outset as a reproducible teaching instrument for embedded edge AI. Specifically, it integrates deterministic MCU control, NPU-accelerated inference, protected multi-source power delivery, vision-oriented high-speed interfaces, modular expansion, and a progressive curriculum framework into a unified platform. This transforms commercially available components and mature software tools into a validated educational system that supports full-stack learning from peripheral configuration and RTOS scheduling to INT8 model deployment and real-time edge inference. The key difference from conventional development boards, then, is not what components are used, but how they are organized around teaching needs—with a system architecture validated to connect low-level embedded practice directly to deployable edge AI.

This study does not aim to prove that the proposed platform has been exhaustively validated across all possible embedded edge-AI workloads. Instead, the present manuscript focuses on the engineering integration and pedagogical deployment of the STM32N6 chip in an undergraduate teaching and competition-training context. The PEPoseNet-based pull-up recognition system is used as a representative vision-oriented case study because it simultaneously involves camera acquisition, NPU-accelerated inference, real-time control logic, display feedback, and student-oriented project assessment. Therefore, the conclusions of this paper should be interpreted within the scope of platform construction, curriculum integration, and representative vision-based edge-AI validation. Broader validation across image classification, object detection, speech recognition, anomaly detection, and sensor-based AI workloads will be addressed in future work.

### 1.1. Functionality and Features

This project is built upon the STM32N6 heterogeneous computing platform. By integrating a dedicated NPU acceleration unit and a multi-power domain architecture, it achieves real-time pull-up motion analysis at the edge. Its core functionalities include: employing the independently designed lightweight dual-branch network PEPoseNet (incorporating a Heatmap-Keypoint collaborative architecture and gradient freezing strategy) to accurately locate 13 human body keypoints and 2 horizontal bar keypoints (achieving 83.8% PCK@0.2); utilizing depthwise separable convolutions to compress the model size, enabling efficient inference on the STM32N6 at 32 FPS for 512 × 512 resolution inputs; and integrating a five-state motion model (Preparation/Hanging/Ascent/Arrival/Descent) with spatiotemporal feature extraction (e.g., joint angles, relative distances). Action state recognition (99.8% accuracy) and erroneous movement detection (e.g., 99.0% recognition rate for leg bending) are realized through a random forest classifier and mode filtering. The system characteristics are as follows: at the hardware level, a 4-layer PCB optimizes signal integrity, supporting MIPI-CSI camera capture and LCD real-time feedback (with an expected latency target of <31 ms, empirically verified as 18.4 ms); at the software level, a custom PULL-MARKER semi-automatic annotation tool was developed to build the specialized dataset SDUST-PUT (containing over 2000 samples). Ultimately, an end-to-end automated assessment closed-loop is achieved, significantly improving evaluation efficiency compared to manual counting while maintaining measured power consumption below the 3 W design budget.

### 1.2. Key Technical Features

Intelligent pull-up assessment at the edge is achieved through co-optimization of the heterogeneous architecture and the algorithm stack.

Hardware: The STM32N6 processor pairs a Cortex-M55 core with a 600-GOPS dedicated NPU, balancing control determinism with AI throughput. A custom multi-power-domain design—SMPS for high-transient core loads and LDOs for noise-sensitive I/O with <50 mV ripple—supports real-time visual processing while keeping total dynamic power under a 3 W thermal envelope.

Algorithm: The lightweight dual-branch network PEPoseNet uses an improved hourglass structure in its heatmap branch to generate 15-point confidence maps. The keypoint branch applies gradient freezing and intermediate-layer feature reuse to avoid information loss at the final layers. Joint loss optimization yields 83.8% PCK@0.2 keypoint accuracy, and depthwise separable convolutions compress the model to run at 32 FPS on device.

Application: A spatiotemporal assessment model defines a five-state motion cycle (Preparation/Hanging/Ascent/Arrival/Descent). From 2270-dimensional spatiotemporal features—joint angles, relative distances, and similar measures—a random forest classifier is trained. Mode filtering suppresses state misjudgments, giving 99.8% motion-state recognition and 99.0% error detection, forming a complete capture-localization-assessment edge-intelligence pipeline.

### 1.3. Main Contributions

The main academic and engineering contributions of this work are summarized below:

(1) Educational hardware design based on STM32N6. We designed and fabricated a modular STM32N6-based system board for embedded edge-AI teaching. The board integrates MIPI-CSI vision input, RGB display output, protected dual-input power delivery, multi-domain voltage regulation, high-speed memory, and an 80-pin expansion connector. The STM32N6 series integrates a custom Neural-ART Accelerator delivering 0.6 TOPS, marking ST’s first MCU with an in-house NPU and enabling efficient real-time edge inference [12]. Compared with general-purpose evaluation boards, the proposed design emphasizes repeated student use, protected power connection, quick peripheral replacement, and vision-oriented AI deployment.
(2) Reproducible edge-AI teaching workflow. We also provide an end-to-end educational pipeline spanning dataset construction, semi-automatic keypoint annotation, lightweight model training, INT8 quantization, STM32Cube.AI operator mapping, and on-device NPU inference. The entire workflow is reproducible by both students and instructors, bridging algorithm learning with hands-on embedded deployment practice.
(3) Algorithm–hardware–curriculum co-design. A lightweight dual-branch pose estimator, PEPoseNet, is mapped to the STM32N6 NPU and packaged as a curriculum-focused pull-up recognition case study. The model is not intended as a general-purpose state-of-the-art pose estimator; instead, it serves as a hardware-executable educational payload that illustrates the trade-offs among accuracy, latency, memory footprint, and power under MCU-class edge-AI constraints. Because the application-level validation in this paper is anchored to this representative vision-based teaching case, broader generalization to other AI workloads is not claimed as a completed result but is noted as a key direction for follow-up work.

The remainder of this paper is organized as follows. Section 2 presents the advantages of Tiny Machine Learning (TinyML) and STM32N6 platform. Section 3 introduces the overall architecture of the proposed STM32N6-based embedded edge-AI teaching platform and its curriculum-oriented design, as well as design of the improved PEPoseNet algorithm and its deployment-oriented considerations. Section 4 describes the implementation of the pull-up recognition system and the integration of the algorithm with the hardware platform. Section 5 reports the experimental results and comparative analysis. Finally, Section 6 concludes the paper and discusses future work.

## 2. Related Work

To frame the contribution of this work, this section reviews recent progress in TinyML, hardware acceleration architectures, and edge-AI educational platforms, then establishes a comparative architectural taxonomy.

### 2.1. Evolution of TinyML Systems and Embedded AI

Tiny Machine Learning (TinyML) has shifted AI inference from cloud-centric architectures toward resource-constrained microcontrollers (MCUs) [13]. Recent work has explored algorithmic co-design extensively, drawing on frameworks such as TensorFlow Lite for Microcontrollers (TFLite Micro) and CMSIS-NN, paired with techniques including INT8 quantization and structural pruning [14]. Nonetheless, running highly parallel matrix operations on conventional scalar processors—such as standard ARM Cortex-M4/M7 cores [15,16]—leads to prohibitive inference latency and elevated energy consumption per Multiply–Accumulate (MAC) operation [17]. This fundamental hardware–software mismatch motivates a shift toward on-chip Neural Processing Unit (NPU) integration.

### 2.2. Hardware Acceleration Architectures for Edge AI

To overcome the computational limits of standard MCUs, several real-time MCU/NPU deployment platforms have emerged in recent years.

Application-Specific AI Chips (e.g., Kendryte K210, MAX78000). These deliver ultra-low-power vision inference through dedicated CNN accelerators, but their highly proprietary toolchains and rigid memory topologies raise the learning curve for undergraduates and limit flexible peripheral expansion.

High-End Crossover MCUs (e.g., NXP i.MX RT series). These devices offer substantial computational headroom, yet they typically lack vendor-supported quantization toolchains streamlined for pedagogical use.

By comparison, the STM32N6 adopts a heterogeneous architecture (Cortex-M55 + embedded 600-GOPS Neural-ART NPU) backed by the mature STM32Cube.AI ecosystem. It therefore achieves MPU-level AI throughput while retaining the deterministic control and open educational ecosystem characteristic of standard ARM-based MCUs.

### 2.3. Educational Edge AI Kits and Deployment Frameworks

In embedded systems education and technical competitions, existing teaching platforms generally fall into three architectural categories:

High-Performance MPUs (e.g., Raspberry Pi 4, NVIDIA Jetson Nano). These currently dominate AI prototyping thanks to GPU/multi-core capabilities and mature Linux ecosystems. Limitation: high power consumption (>5 W), thermal throttling, and non- predictable scheduling make them less suitable for battery-powered, hard-real-time robotic control tasks [18].

Conventional IoT MCUs (e.g., ESP32-S3). Widely used in low-cost embedded educational kits, they excel at wireless connectivity and basic control. Limitation: without a dedicated NPU, their edge vision capabilities are restricted to very low-resolution or non-real-time tasks.

High-End MCU Educational Kits (e.g., STM32F4/H7 series, Arduino Portenta H7). These robust microcontrollers are traditionally used in embedded education for real-time deterministic control and sub-1 W power profiles [19]. Limitation: lacking hardware neural acceleration, they cannot mathematically achieve >30 FPS on complex vision tasks such as pose estimation within strict thermal constraints.

Compared with the above platforms, the STM32N6 offers a compromise between MCU-level deterministic control and hardware-accelerated AI inference. It retains the control-oriented development workflow of traditional MCU systems [19], while providing NPU support for real-time vision workloads within a low-power embedded design. This makes it suitable for teaching scenarios in which students need to learn both peripheral-level programming and edge-AI deployment.

### 2.4. Difference from Existing STM32 Development Boards and Educational Kits

Although a number of STM32-based development boards and educational kits are available, their design objectives differ substantially from those of the proposed platform. Official STM32 evaluation and discovery boards are primarily intended to expose the functional capabilities of a specific microcontroller family, including clock configuration, peripheral access, and vendor toolchain demonstration. They are highly useful for chip evaluation and low-level embedded learning, but they are not necessarily optimized for repeated student competition use, protected power reconfiguration, vision-centered AI workloads, or a complete educational workflow from sensor acquisition to NPU inference and result feedback.

Traditional STM32F4/H7 educational boards emphasize deterministic control, low-power operation, and peripheral programming. However, because they do not contain a dedicated neural processing unit, they cannot provide real-time vision inference for moderately complex models such as pose estimation without severe reductions in input resolution or model complexity. Conversely, high-level AI educational kits such as Jetson Nano or Raspberry Pi-based systems provide stronger software ecosystems and higher-level AI prototyping convenience, but they tend to obscure low-level embedded constraints such as memory layout, power budgeting, interrupt-driven control, and real-time scheduling.

The proposed platform is fundamentally different in three aspects. Initially, it is organized around heterogeneous MCU–NPU cooperation, where the Cortex-M55 core is reserved for deterministic control and peripheral orchestration, while the Neural-ART NPU executes INT8 neural inference. Moreover, the board-level design introduces student-oriented reliability, including dual-input power switching, reverse-polarity protection, reverse-current blocking, overcurrent limiting, soft-start control, and modular expansion through an 80-pin connector. These features are not merely auxiliary circuits; they directly address common failure modes in open-ended student prototyping. Lastly, the hardware is coupled with a structured teaching framework and a reproducible deployment pipeline, enabling students to experience the complete chain of embedded edge AI, including sensor interfacing, RTOS scheduling, quantization, NPU mapping, and application-level evaluation. Therefore, the proposed contribution should be understood as a system-level educational platform rather than a conventional microcontroller demonstration board.

## 3. Materials and Methods

**Figure 1:**
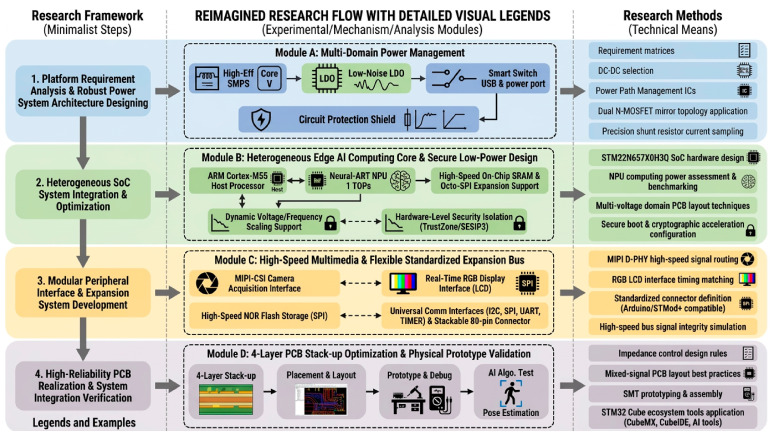
Overall Architecture of the Proposed STM32N6-based Teaching Platform. The architecture is divided into three main layers: the hardware foundation featuring the heterogeneous STM32N6 SoC, the software and AI toolchain for INT8 quantization, and the application layer demonstrating the end-to-end pull-up recognition task. Blue arrows represent data flow, while red dashed arrows indicate control signals.

This section begins with the overall framework of the proposed system, as shown in Figure 1, to provide a concise overview of its architecture, core modules, and technical implementation path. The framework integrates requirement analysis, heterogeneous SoC optimization, modular interface expansion, and PCB realization and verification. It illustrates the complete development process of the modular embedded edge AI platform.

The board was designed according to the requirements observed in embedded edge-AI teaching and student competitions. Unlike a general evaluation board that mainly exposes chip functions, this platform gives priority to camera input, display feedback, protected power supply, and stable expansion interfaces. These functions are frequently used in student projects involving visual perception, real-time control, and on-device inference. Therefore, the hardware design focuses on four aspects: separation of control and inference tasks, safe power connection during repeated experiments, high-bandwidth image acquisition, and modular access to external sensors and actuators.

Current technological competitions for electronics and information science undergraduates impose the following requirements on embedded edge AI teaching platforms:

(1) Strong Computational Acceleration Capability: Integration of a dedicated NPU (Neural Processing Unit) to support efficient model deployment and computational acceleration, meeting the demands of real-time and high-precision scenarios.
(2) Abundant Image Acquisition Interfaces: Configuration of MIPI-compliant CSI camera interfaces, ensuring compatibility with mainstream embedded devices (e.g., Raspberry Pi, Jetson series) and the mobile terminal hardware ecosystem (e.g., smartphones, tablets).
(3) Visual Interactive Functionality: Provision of an RGB color screen interface to support real-time image display and interactive operation.
(4) Stackable Modular Design: Adoption of a stackable hardware architecture that allows for flexible combination and tailoring of functional modules, adapting to diverse practical training scenarios such as teaching, competitions, and project development.
(5) Broad Power Compatibility: Inclusion of a standard USB power supply interface, compatible with common power sources like laptops and power banks, ensuring convenience for teaching and practical activities.
(6) Comprehensive Expansion Interfaces: Integration of communication interfaces including I2C, SPI, UART, and TIMER, enabling the connection of various sensors and covering application domains such as industrial intelligence, smart home, and embodied AI [20,21,22].

**Figure 2:**
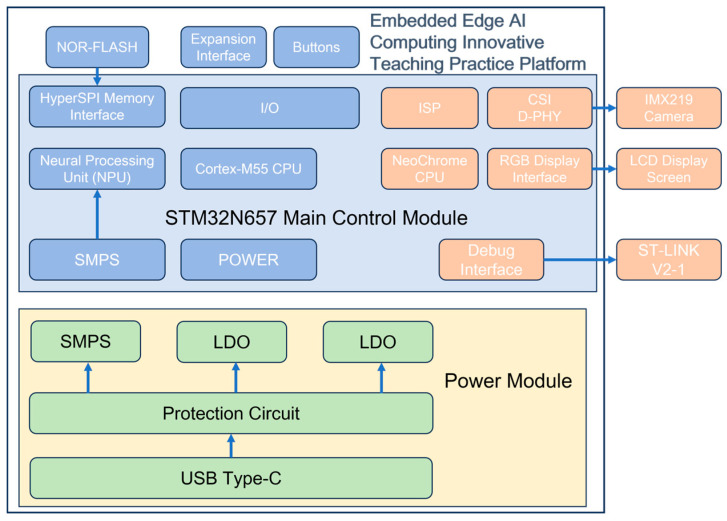
Functional Diagram of the Proposed Platform.

Based on the aforementioned requirements, this paper proposes an overall architectural design of the proposed platform, as illustrated in Figure 2. The platform centers around the STM32N657X0H3Q chip (STMicroelectronics, Geneva, Switzerland) and integrates modules for high-speed memory, high-speed camera interfaces, RGB display interfaces, keypad input, expansion interfaces, and power management.

### 3.1. Modular Hardware Platform Design

#### 3.1.1. Design of the Power Supply Circuit

**Figure 3:**
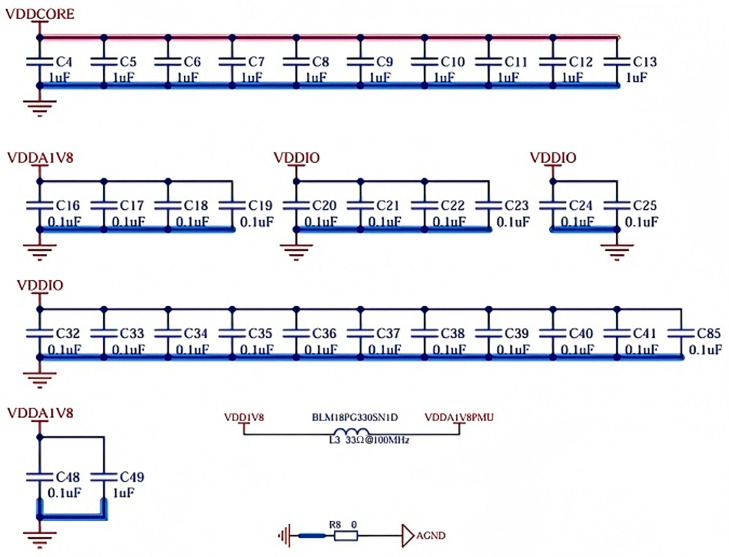
Schematic diagram of the multi-power-domain supply architecture. The circuit integrates SMPS for high-transient core loads and Low-Dropout regulators (LDOs) for noise-sensitive I/O domains. The dual N-MOSFET mirror circuit at the input terminal ensures reverse-polarity protection and soft-start control.

The power subsystem was designed for low-power operation and repeated classroom use. The platform uses separate power domains for the STM32N6 core, I/O circuits, camera module, display module, and expansion interfaces. Switched-mode regulators supply the high-current domains, while low-noise LDO regulators are used for I/O and noise-sensitive circuits [23]. This arrangement improves conversion efficiency and helps reduce supply ripple on peripheral interfaces. The architecture is shown in Figure 3.

The platform supports automatic switching between USB power and an external power input. A power-path management circuit monitors the two inputs and selects the available source according to the voltage condition. When USB power is removed or falls below the threshold, the system switches to the external input within the measured switching time. During this process, input capacitors help maintain the core supply voltage and reduce the risk of reset. This function is useful in teaching experiments because students often move between USB debugging and battery-powered demonstrations.

Reverse-current and reverse-polarity protection are included at the power input. In student experiments, reverse connection, mixed USB/external supply, and peripheral pre-powering may occur. These conditions can cause current to flow back through protection diodes or I/O paths, which can increase the risk of latch-up or device damage. To reduce this risk, the input stage uses a dual N-MOSFET protection structure. Under normal polarity, the MOSFETs provide a low-resistance conduction path. Under reverse polarity or reverse-current conditions, the structure blocks the current path and isolates the downstream circuits.

The mirror circuit designed for this platform adopts a dual N-MOSFET symmetric structure: when the power is correctly phased, the MOSFETs are forward-biased and conducting, ensuring a low-impedance power supply pathway; when reverse current is detected, the body diodes of the MOSFETs are reverse-biased and cut off, forming a physical isolation barrier to block the reverse current path. For reverse power connection, the mirror circuit’s symmetric topology directly severs the entire power path, while a built-in reverse voltage detection diode triggers an alarm, preventing irreversible damage to critical components such as the microcontroller and NPU. To enhance protection reliability, the PCB layout is topologically optimized: the power and signal paths are physically separated, and the mirror circuit is positioned strictly adjacent to the power input interface to minimize the reverse current propagation loop.

It also integrates electronic current-limiting switches to provide overcurrent protection and soft-start control. That also improves system reliability and electrical safety [24]. The electronic current-limiting switch realizes overcurrent protection through a strictly defined “current sampling + threshold comparison + rapid response” mechanism: a precision shunt resistor in the power path converts the load current into a voltage signal, which is fed into the IC’s internal comparator. This architectural implementation not only protects the power management chip and core components from destructive inrush currents but also minimizes voltage fluctuation impacts on sensitive peripheral domains (such as cameras and LCD screens), ensuring the deterministic initialization of edge AI algorithms and peripheral modules. From an engineering perspective, these protection functions significantly enhance the platform’s fault tolerance against dynamic peripheral hot-swapping and unverified load conditions inherent in rapid prototyping. The overcurrent protection instantly isolates physical faults, while the soft-start control dampens startup transients when multiple peripherals are connected simultaneously. This design scientifically balances hardware flexibility with the practical reliability required for student competition environments.

In student-led engineering projects, these protection functions improve the platform’s fault tolerance: students may connect mismatched peripherals or have wiring errors during the intense prototyping phase. The overcurrent protection function can quickly cut off the power to avoid equipment damage, while the soft-start control ensures stable startup even when multiple peripherals are connected simultaneously. This design balances the flexibility of the educational platform and operational safety, reducing maintenance costs and ensuring the smooth progress of laboratory instruction and practical evaluations. Compared with the power design of general-purpose STM32 evaluation boards, the proposed power architecture is deliberately over-specified for educational robustness. In student projects, incorrect polarity connection, simultaneous USB and battery supply, peripheral hot-plugging, and short-term overcurrent events occur much more frequently than in professional laboratory evaluation. For this reason, the power subsystem is treated as an educational reliability module rather than a simple voltage-conversion circuit. This design choice is essential for reducing hardware failure rates during intensive training and for allowing students to focus on algorithm deployment and system integration instead of board recovery and fault repair.

#### 3.1.2. SoC System Design

**Figure 4:**
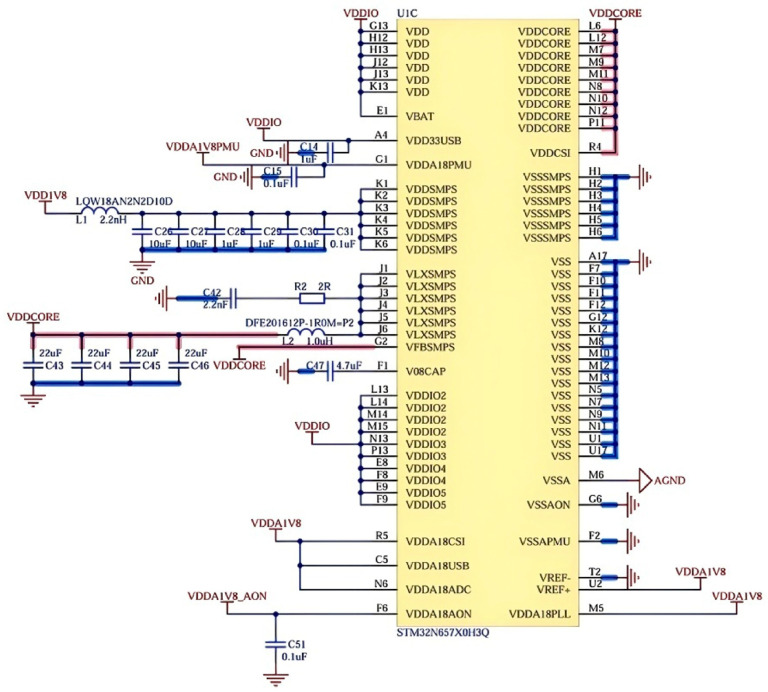
Detailed schematic of the STM32N657X0H3Q system-on-chip (SoC) integration. This schematic highlights the connections between the Cortex-M55/NPU core and critical high-speed peripherals, including the MIPI-CSI interface for vision acquisition and the Octo-SPI Flash interface for memory expansion. Decoupling capacitors and local power nets are optimized to support the 600-GOPS NPU dynamic workload.

To achieve more efficient edge AI computing, the system-on-chip (SoC) must possess high-performance computing capabilities, large-capacity and efficient memory, rich peripheral interfaces, and low-power design. Therefore, this platform employs the STM32N657X0H3Q as its core component, as shown in Figure 4. This high-performance MCU from STMicroelectronics is tailored for embedded edge-AI computing. According to the official STM32N6 series documentation released by STMicroelectronics, the device integrates an Arm Cortex-M55 core (STMicroelectronics, Geneva, Switzerland) operating at up to 800 MHz, a Neural-ART Accelerator running at up to 1 GHz with a peak performance of 600 GOPS, and 4.2 MB of contiguous embedded SRAM [25]. These officially specified hardware characteristics, together with the available high-speed peripheral interfaces such as MIPI-CSI, RGB display, Octo-SPI, SPI, I2C, UART, and TIMER resources, provide the basic architectural foundation for deploying lightweight vision models on an MCU-class platform. In this work, these official hardware specifications are used as the reference for processor capability, memory resources, and interface availability, while our experimental evaluation focuses on system-level latency, power consumption, stability, and applicability for embedded edge-AI education after board-level integration. Its multi-power domain design ensures ultra-low power consumption (suitable for battery-powered setups) and hardware-level security (Arm TrustZone, SESIP3 certification) protects project codes and data, while the mature STM32 Cube ecosystem (STM32CubeMX, STM32CubeIDE, ST Edge AI tools) simplifies hardware configuration, model conversion, and program debugging—helping students bypass low-level development and focus on algorithm deployment, significantly shortening competition project cycles.

The STM32N657X0H3Q was selected because its architecture matches the requirements of the proposed teaching platform. First, the Cortex-M55 core can handle RTOS scheduling, peripheral management, and control logic, while the Neural-ART NPU executes INT8 neural-network inference. This separation helps reduce interference between control tasks and inference tasks. Second, the 4.2 MB on-chip SRAM encourages students to consider model size, tensor buffers, and memory placement during deployment. In this work, PEPoseNet is compressed to fit within the available memory resources, reducing dependence on external memory access during inference. Third, the device provides interfaces such as MIPI-CSI, RGB display, SPI, I2C, UART, TIMER, and Octo-SPI, which are commonly required in vision-based competition projects.

#### 3.1.3. Peripheral and Interface Design

**Figure 5:**
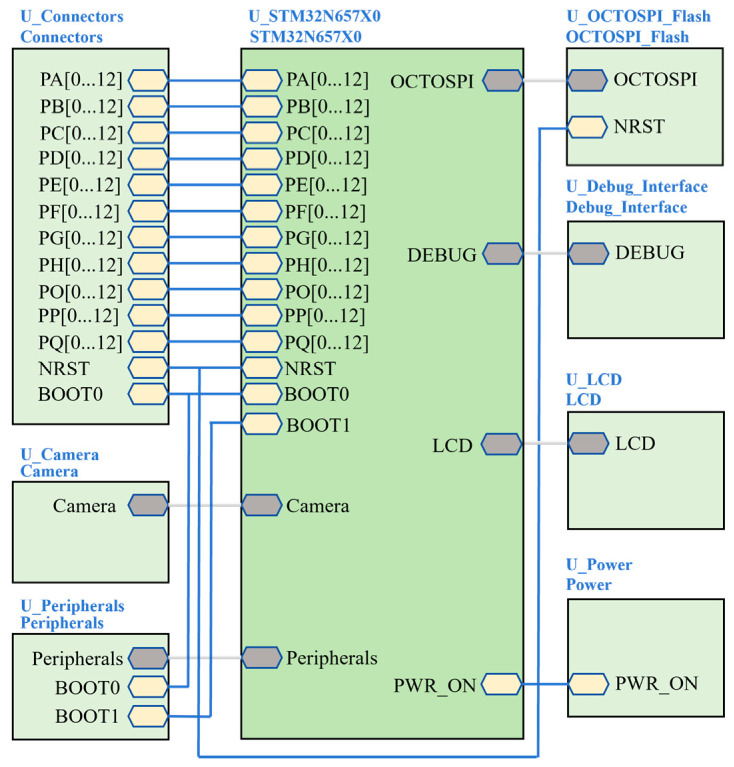
Schematic Diagram of Peripheral Devices and Interface Design.

Given that the technological competitions university students participate in typically feature open-ended topics, the platform needs to provide a rich set of peripherals and interfaces to accommodate the flexibility and diversity of student project themes. Accordingly, the platform integrates buttons, high-speed NOR Flash, an MIPI-CSI interface, an RGB display interface, as well as abundant expansion interfaces including SPI, I2C, UART, and TIMER. Furthermore, the platform utilizes an 80-pin board-to-board connector to bring out unused I/O, offering highly flexible expansion capabilities, as illustrated in Figure 5. Standardized modular interface design can improve the compatibility of embedded platforms with peripheral devices, which is an important design principle adopted by this platform [26].

This figure presents a modular connection block diagram between the platform’s core control unit (left side) and extended peripherals (right side), clearly mapping the interface allocation logic that supports scalable system development. For instance, the MIPI-CSI interface serves as the primary high-bandwidth data ingestion node (e.g., for the pull-up motion quality assessment system mentioned in earlier sections). Developers can connect a camera through this interface to stream raw visual data via direct memory access (DMA), thereby leveraging the platform’s edge AI computing power for real-time pose recognition without overburdening the CPU’s polling cycles.

The “Peripheral” module orchestrates the SPI, I2C, UART, and TIMER interfaces, matching various specialized edge nodes: SPI interfaces connect to high-speed NOR Flash, providing sufficient and non-volatile storage space for massive edge AI model weights or local tensor datasets; I2C interfaces support low-power compact OLED displays for state monitoring; UART interfaces enable asynchronous connections to Bluetooth or telemetry modules; TIMER interfaces output highly precise PWM signals, driving motors for closed-loop robotic arm or autonomous vehicle control, which are essential in dynamic embedded applications.

The on-board buttons are connected to GPIO pins and can be used for mode switching, reset control, and simple human–machine interaction. The NOR Flash connected through SPI provides non-volatile storage for model parameters, test data, or configuration files. By exposing common interfaces through the 80-pin connector, the platform allows students to add sensors, wireless modules, motor drivers, or other competition-specific peripherals without redesigning the core board. This modular interface design reduces wiring complexity and improves the repeatability of laboratory experiments.

#### 3.1.4. PCB Design

**Figure 6:**
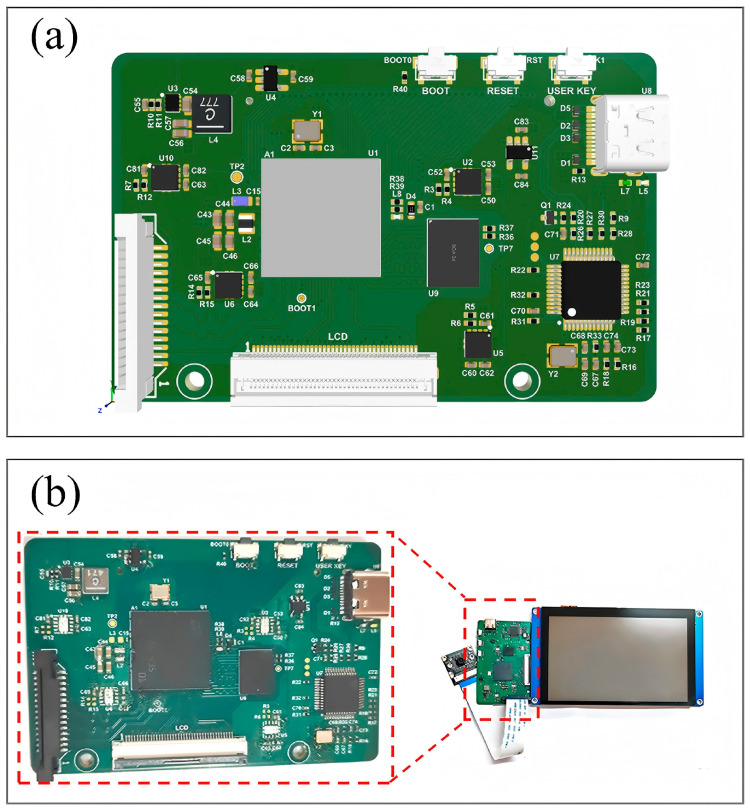
PCB Design Drawings and Physical Diagrams: (**a**) PCB Design Drawings; (**b**) Physical Diagrams.

The platform uses a four-layer PCB stack-up consisting of a top signal layer, a solid ground layer, a power layer, and a bottom signal layer. The continuous ground plane provides a stable return path for high-speed signals and reduces loop inductance, electromagnetic interference, and crosstalk. This layout is important for MIPI-CSI image acquisition, RGB display output, external memory access, and DMA-based data transfer during edge-AI inference. The PCB layout and the fabricated board are shown in Figure 6.

Figure 6a shows the detailed PCB layout of the core control board, designed to meet both high-frequency signal integrity and hardware abstraction requirements. The central chip (U1) is the STM32N6 high-performance microcontroller—the platform’s core processing unit—with supporting components placed around it. To suppress transient power noise during high-throughput NPU operation, passive components (capacitors C28–C31, resistors R12–R16) are positioned for optimal power filtering and signal conditioning. Crystal oscillators Y1 and Y2 provide stable, low-jitter clock signals. Practical interfaces—including a USB port (U8) for programming and data transfer, an LCD connector for display expansion, and user buttons (BOOT, RESET, USER KEY)—are physically separated to avoid mutual interference. The layout follows a strict four-layer stack-up (Top Signal–Solid GND–Power Plane–Bottom Signal), which effectively reduces electromagnetic interference (EMI) and crosstalk.

Figure 6b presents the physical implementation of the platform. The red dashed box marks the assembled core PCB, corresponding to the layout in Figure 6a; extended modules (LCD screen and camera) connect through dedicated high-bandwidth interfaces. This modular physical topology is optimized for rapid iterative prototyping rather than serving purely as a demonstration board: the core computing domain is physically isolated from unvalidated peripheral inputs. Developers can therefore debug hardware drivers and test complex edge-AI algorithms such as human pose estimation independently, without risking core system stability. This design validates the full workflow from embedded hardware design to edge-AI application deployment, while ensuring the electrical stability needed for continuous edge-AI computation and providing a robust, fail-safe foundation for complex application deployment.

#### 3.1.5. Engineering Achievements

**Figure 7:**
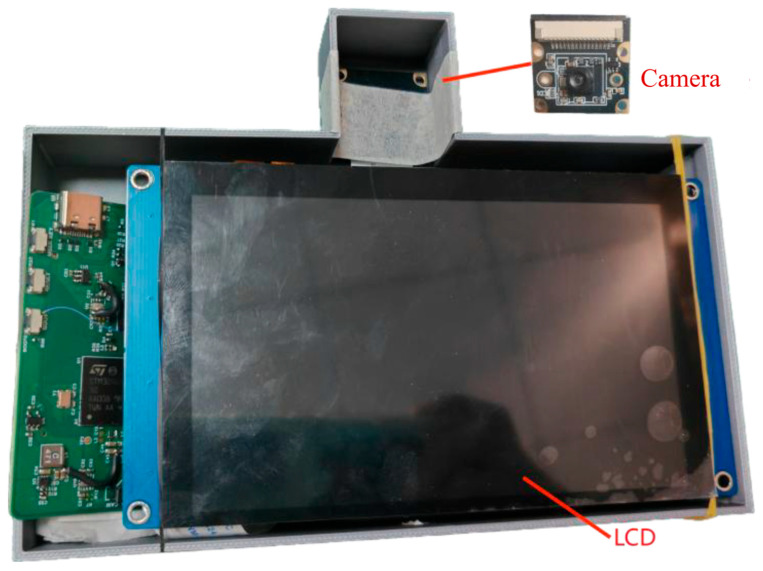
Physical implementation of the STM32N6 platform. Schematic layout highlighting component placement and thermal design considerations. Front view of the fabricated physical prototype, showing the modular stack-up structure with the integrated LCD screen and MIPI-CSI camera module.

Figure 7 presents the final fabricated results, showing the Schematic Diagram of the STM32N6 System Board and the Front View of the Physical Prototype, respectively.

### 3.2. Edge AI Algorithm and Deployment Workflow

Meeting real-time requirements for edge vision applications means algorithm design must be closely co-optimized with hardware constraints. This section describes the proposed lightweight vision algorithm and its end-to-end deployment pipeline on the STM32N6 platform.

#### 3.2.1. Design of the Lightweight Vision Model (PEPoseNet)

Standard human pose estimation models are often too computationally heavy for MCU-class devices, particularly in pre-competition training scenarios such as intelligent pull-up recognition. To address this, we adopt PEPoseNet—a dual-branch lightweight network tuned for the STM32N6’s 0.6 TOPS NPU. The architecture is optimized explicitly for edge execution:

Heatmap Branch. An improved lightweight hourglass structure generates 15-point confidence maps, capturing spatial context while keeping memory footprint small.

Keypoint Regression Branch. To avoid the information loss at final layers common in heavily compressed models, this branch uses a gradient-freezing strategy combined with intermediate-layer feature reuse.

Together, the dual-branch design keeps keypoint localization accuracy high while sharply reducing parameter count, making the model well suited for resource-constrained edge inference. For reproducibility in teaching settings, the training configuration is standardized. The network takes a fixed 512 × 512 input and is trained with the Adam optimizer at an initial learning rate of 10^−3^, batch size 32, and cosine annealing over 150 epochs.

#### 3.2.2. AI Toolchain and Quantization Pipeline

The deployment process was organized as a teaching workflow that connects model training with embedded implementation. First, PEPoseNet is trained on a PC and exported to a TensorFlow Lite-compatible format. Second, post-training quantization is applied to convert weights and activations to INT8. A representative calibration set of 100 randomly sampled training images is used to determine activation scales and zero-points. Third, the quantized model is imported into STM32Cube.AI, which generates C code and maps supported operators to the Neural-ART NPU. The generated files are then integrated into the STM32CubeIDE project for on-device inference.

#### 3.2.3. End-to-End Edge Inference Data Flow

From a deployment standpoint, the model sits within a deterministic end-to-end embedded pipeline: Sensor → AI Inference → Result Output. The STM32N6 heterogeneous architecture orchestrates this flow efficiently:

(1) Pre-processing (Cortex-M55). High-speed camera interfaces capture raw frames. The ARM Cortex-M55 core handles resizing, normalization, and memory formatting to prepare input tensors.
(2) Hardware-Accelerated Inference (NPU). Pre-processed tensors are passed to the NPU, which runs PEPoseNet inference in parallel, achieving millisecond-level latency without blocking main control tasks.
(3) Post-processing and Control (Cortex-M55). The M55 core reads output tensors (confidence maps and coordinates) from the NPU, applies non-maximum suppression (NMS) and logic such as pull-up counting, and drives the RGB display and peripherals to output results in real time.

This structured workflow validates PEPoseNet not only at the model level but also within a working embedded system, providing a complete template for student project development.

### 3.3. Pedagogical Framework and Curriculum Design

To build students’ practical skills in embedded edge-AI computing across different proficiency levels, the platform organizes hands-on training into seven progressive stages: Foundation & Introduction, Sensors & Communication, Real-Time Operating System, Edge AI Fundamentals, AI Model Deployment, Comprehensive Application, and Project Assessment. Students start with basic STM32 peripheral operations, move through RTOS multi-task programming and AI model deployment, and ultimately reach integrated application capability. Converting and optimizing AI models with STM32Cube.AI substantially reduces resource usage while preserving inference accuracy [27]. Detailed training projects and their hour allocations are listed in Table 1.

The practical training content arrangement and design in Table 1 closely align with the actual needs of embedded edge AI competition teaching. Its core significance and benefits are reflected in the deep integration of teaching logic, practical adaptability, and competition ability cultivation “Foundation & Introduction → Sensors & Communication → Real-Time Operating System → Edge AI Fundamentals” The stage division follows the cognitive progressive law from hardware foundation to software development and then to AI technology. It not only avoids the beginners’ fear of directly encountering complex edge AI technology, but also lays a solid practical foundation for subsequent sensor fusion and multi-task scheduling through basic experiments such as GPIO control and UART communication. The duration allocation of 2 to 3 h for each stage is adapted to the focused pace of offline training, ensuring the depth of practical operation for individual skills (such as I2C/SPI driver development). Furthermore, through the cohesion of the content (such as learning from the TensorFlow Lite Micro framework to the deployment of the STM32Cube.AI toolchain), a complete knowledge chain of “hardware control–algorithm development–system integration” has been constructed; The “Objectives and Skills” column clearly defines the quantitative learning goals for each stage, helping students focus on core competencies (such as model compression and end-to-end system development). Finally, the defense and code review designs in the “Comprehensive Application” and “Project Assessment” sections are directly benchmarked against the project presentation and engineering specification requirements of science and technology competitions. While consolidating embedded edge AI technology, it has cultivated students’ abilities in teamwork, presentation of achievements and engineering thinking.

To better accommodate students with different proficiency levels, the seven training stages are organized into three progressive difficulty tiers, as summarized in Table 2. Each stage is further associated with measurable completion criteria, allowing instructors to objectively assess student progress and to recommend personalized learning trajectories.

Based on this tiered structure, three typical learning trajectories are recommended:

Beginner trajectory (Stages 1–2): Students with no prior embedded development experience focus on fundamental peripheral programming and sensor communication. Upon completing these two stages, they are expected to independently configure basic STM32 peripherals and implement simple data acquisition tasks.

Advanced trajectory (Stages 1–4): Students who have completed basic embedded coursework proceed through sensor communication, RTOS programming, and edge AI fundamentals. After Stage 4, they should understand the complete edge AI workflow from model training to quantization, although they may not yet deploy models independently.

Competition trajectory (Stages 1–7): Students preparing for embedded AI competitions complete all seven stages. The final project assessment stage serves as a summative evaluation, where students must demonstrate a fully functional edge AI system, defend their design choices, and pass a code review.

This layered structure meets the needs of learners at all levels, benefiting both beginners encountering embedded systems for the first time and students preparing for national competitions. At the same time, it provides teachers with clear and observable assessment criteria to measure learning progress at each stage.

## 4. Results

### 4.1. Evaluation Methodology

To clarify the measurement context of the reported latency, FPS, power consumption, and accuracy, the main evaluation settings are summarized here. All hardware tests were conducted on the fabricated STM32N6-based prototype at an ambient temperature of 25 °C. The STM32N657X0H3Q was configured with the Arm Cortex-M55 core running at up to 800 MHz. The Neural-ART Accelerator was enabled for INT8 inference. The system used the high-performance clock configuration generated by the STM32Cube toolchain.

The embedded software was developed using the STM32Cube ecosystem, including STM32CubeMX, STM32CubeIDE, STM32Cube.AI, and the STM32CubeN6 firmware package. The STM32CubeN6 firmware package used in this work was version 1.0.0. The complete project configuration files are provided in the GitHub repository to support reproducibility.

For the pull-up recognition application, image frames were acquired through the MIPI-CSI camera interface. DMA-assisted frame transfer was used to reduce CPU blocking during image acquisition. Each frame was resized or cropped to 512 × 512 before being fed into PEPoseNet. The RGB display was used for real-time feedback. The reported 32 FPS value corresponds to the complete live application pipeline, including camera acquisition, DMA transfer, preprocessing, NPU inference, post-processing, motion-state judgment, and display feedback. Moreover, the deployed PEPoseNet model used post-training INT8 quantization through STM32Cube.AI. Both weights and activations were quantized to 8-bit integers.

In this paper, two latency metrics are reported. The first is inference-only latency, measured with a pre-loaded 512 × 512 input tensor in memory. The timer starts when the tensor is passed to the inference function and stops when the output tensor is generated. Under this setting, PEPoseNet-INT8 achieves a latency of 18.4 ms, corresponding to 54.3 FPS. The second metric is full-pipeline latency, which includes camera acquisition, DMA transfer, preprocessing, NPU inference, post-processing, motion-state judgment, and display update. The complete application pipeline achieves 31.3 ms per frame, corresponding to 32.0 FPS.

### 4.2. Controlled Environment Performance Assessment

Before evaluating performance in open-ended competition settings, we first tested the platform under tightly controlled conditions to characterize its intrinsic capabilities. Competition outcomes alone are confounded by variables such as student skill level, environmental noise, and inconsistent project requirements, which obscure the platform’s own behavior. We therefore established a standardized benchmark protocol.

Experimental Setup: The platform was isolated in a controlled thermal environment (25 °C). The power consumption was monitored using a high-precision digital power analyzer (e.g., Keysight N6705C) at the SMPS and LDO domains. To stress-test the heterogeneous architecture (0.6 TOPS NPU + CPU), standard quantization models (INT8/FP16) from the MLPerf Tiny benchmark suite were deployed. For clarity, the “System Idle” condition in the benchmark table refers to a firmware baseline mode rather than a full operational vision mode. In this mode, the system firmware had completed initialization, the main clock generators and required PLLs were enabled, and the RTOS idle task was running. However, the RGB LCD backlight was turned off, the camera module was not streaming image frames, the MIPI-CSI data path was inactive, and the Neural-ART NPU was clock-gated or placed in a low-power idle state. No external expansion modules were connected during this measurement. Therefore, the idle value should be interpreted only as the baseline board-level consumption after initialization, not as the power consumption of the complete pull-up recognition application.

Quantitative Metrics: Table 3 presents the empirically measured end-to-end inference latency, throughput (FPS), RAM/Flash utilization, and peak dynamic power across different load conditions.

Performance Stability: A 72 h continuous workload test was also conducted using the INT8 PEPoseNet inference task. During the test, the board remained operational, and the measured input power stayed below 3.0 W. Serial logs were used to monitor task execution and software-reported CPU/NPU status. This test indicates that the current hardware design is stable for long-duration teaching demonstrations and competition-style workloads. However, it does not replace detailed physical-layer validation such as power-ripple measurement, thermal imaging, or high-speed signal-integrity testing.

This controlled assessment isolates the hardware–software co-optimization benefits, providing a reproducible baseline that confirms the platform provides sufficient computational headroom and stability for subsequent complex, unpredictable competition deployments.

### 4.3. Comparative Statistical Analysis with Alternative Platforms

To quantitatively characterize the performance and energy-efficiency trade-off of the proposed STM32N6 platform under a controlled benchmark setting, we conducted a statistical comparison against two representative platforms widely used in university technology competitions. We selected the Raspberry Pi 4B (representing high-performance, higher-power MPUs) and the STM32H743 (representing traditional high-end MCUs without a dedicated NPU) as baseline counterparts.

To ensure a fair, rigorous, and reproducible benchmark, strict control variables were applied across all three platforms.

(1) Same Model and Precision: The exact same fully quantized PEPoseNet model (INT8 precision, converted via TensorFlow Lite) was deployed on all platforms, strictly isolating hardware acceleration capabilities from algorithmic compression benefits.
(2) Same Input Pipeline: To eliminate I/O bottlenecks and camera driver overheads, a static 512 × 512 test image tensor was pre-loaded into the RAM of each platform. The inference timer started exactly when the tensor was fed into the model and stopped upon output generation.
(3) Same Operating Conditions: All tests were conducted in a controlled thermal chamber at 25 °C with the power management policies of the OS/RTOS set to maximum performance mode. To capture true system-level variance and ensure a rigorous experimental design, the evaluation was conducted across M=10 independent experimental sessions. For each session, the devices were cold-booted, and inference was executed for N=1000 continuous iterations. To prevent inflated statistical significance from repeated inference runs, all analyses were conducted at the session level. For each independent session, we first computed the mean latency; the ten session-level means were then pooled to derive the overall mean, standard deviation, 95% confidence interval, and effect size. Treating each cold-boot session as an independent experimental unit yields a more conservative test of latency differences across platforms.

The statistical outcomes, encompassing the aggregated mean latency (μ), standard deviation (σ) derived from all M×N trials, and overall energy efficiency, are summarized in Table 4. It should be noted that this comparison is designed to quantify platform behavior under a specific controlled workload rather than to establish a universal ranking among all embedded AI platforms. The reported results are therefore interpreted within the scope of the identical PEPoseNet-INT8 model, the static 512 × 512 input tensor, and the controlled thermal environment used in this study.

Statistical Validation and Discussion: The comparison shows that the STM32N6 platform has lower latency and smaller latency variation under the tested workload. Its latency is 18.4 ± 0.3 ms, with a narrow 95% confidence interval, indicating stable execution across repeated cold-boot sessions. The Raspberry Pi 4B achieves a mean latency of 45.6 ms but shows larger variation, which may be related to operating-system scheduling and background processes. The STM32H743 has lower power consumption but much higher latency because the model is executed without NPU acceleration. Under this specific PEPoseNet-INT8 benchmark, the STM32N6 achieves 28.31 FPS/Watt, indicating a favorable balance between inference speed and power consumption for the intended teaching scenario.

The proposed STM32N6 platform demonstrates exceptional statistical stability with a latency of 18.4 ± 0.3 ms. The extremely low standard deviation (±0.3 ms) confirms deterministic execution, a critical requirement for real-time control systems in embedded competitions. An independent samples *t*-test based on the aggregated multi-session data between the STM32N6 and Raspberry Pi 4B latency outcomes confirms that the performance improvement is statistically significant (p<0.001). It is crucial to clarify that the source of variation evaluated by this *t*-test primarily stems from underlying architectural and OS-level scheduling differences. For the Raspberry Pi 4B, the variance is driven by the non-deterministic task preemption, background processes, and memory management overhead inherent to the Linux operating system. In contrast, the minimal variance on the STM32N6 is attributed to the strict real-time determinism of the RTOS and the dedicated bare-metal execution pipeline of the NPU. Furthermore, under this specific PEPoseNet-INT8 benchmark, the STM32N6 achieves an energy efficiency of 28.31 FPS/Watt, which is higher than that of the two tested alternative platforms. These results indicate that, for the evaluated workload and experimental conditions, the proposed platform provides a favorable trade-off among computational throughput, execution determinism, and power consumption for edge-AI teaching practices. However, this conclusion should not be interpreted as a universal performance ranking across all possible embedded AI workloads or educational scenarios.

### 4.4. Innovative Practical Activities

To demonstrate the use of the platform in teaching, we developed an intelligent pull-up recognition system as a representative application case. This task was selected because it requires camera acquisition, pose estimation, motion-state recognition, result display, and embedded deployment, which match the main learning objectives of the course. A typical educational case study is the “STM32N6-based Intelligent Pull-up Recognition System.”

This specific case is presented not to claim the platform as a universal real-time vision hardware, but to comprehensively demonstrate its end-to-end deployment workflow—from sensor integration to algorithm execution—in a typical student competition scenario. Accordingly, the experimental results obtained from this case should be interpreted as evidence of the platform’s feasibility for a representative vision-based educational application, rather than as a comprehensive validation of all embedded edge-AI workloads. The purpose of this case study is to verify whether the STM32N6-based platform can support a complete teaching workflow involving data acquisition, model compression, NPU deployment, and real-time feedback within strict MCU-class power and memory constraints. This case addresses the issues of strong subjectivity and low efficiency inherent in traditional manual counting for pull-up tests [28]. Leveraging the STM32N657X0H3Q processor, it utilizes edge computing and computer vision technologies to achieve automated assessment. Its core functionalities include employing a self-designed lightweight dual-branch network, PEPoseNet. For pedagogical reproducibility, the customized dataset, referred to as SDUST-PUT, contains slightly more than 2000 images and was partitioned into an 80:10:10 ratio for training, validation, and testing. The dataset was collected from undergraduate students aged approximately 20–22 years during pull-up training activities. The acquisition was performed in a controlled outdoor environment using a fixed camera position and a standardized horizontal bar. The dataset includes moderate variations in individual posture, execution speed, clothing, and body scale, but the environmental diversity is limited because the background, camera viewpoint, and illumination conditions were intentionally kept relatively stable to support repeatable teaching demonstrations.

This dataset design inevitably introduces potential bias. The participants were limited to university students within a narrow age range, so the dataset does not cover children, elderly users, or a wide range of body types. Additionally, the acquisition environment was controlled and did not include strong illumination changes, complex backgrounds, occlusion, rain, nighttime scenes, or large camera-angle variations. Lastly, the task was restricted to a standardized pull-up scenario, and therefore the reported recognition performance should be interpreted within this constrained educational setting rather than as evidence of general in-the-wild robustness. The annotation specification explicitly defines an object segregation mechanism: it isolates 13 human anatomical joints from 2 static horizontal bar coordinates to separate dynamic motion from static references. The model accuracy was rigorously evaluated using the Percentage of Correct Keypoints (PCK@0.2) metric, where a prediction is deemed correct if the normalized distance falls within 20% of the torso diameter, achieving an overall accuracy of 83.8%, as well as utilizing depthwise separable convolutions to compress the model size, enabling efficient inference on the STM32N6 at 32 FPS for 512 × 512 resolution inputs; and combining a five-state action model (Preparation/Hanging/Ascent/Arrival/Descent) to train a Random Forest classifier.

To rigorously assess the classifier’s reliability, we built a dedicated sequence dataset of 12,500 frame-level samples. The data were split strictly into an 80% training set and a 20% test set, with no subject overlap between the two. The test set (N=2500) has a balanced class distribution: Preparation (18%), Hanging (22%), Ascent (20%), Arrival (19%), and Descent (21%).

On this test set, the classifier reaches 99.8% overall accuracy. It is important to note that this very high accuracy reflects the tightly controlled teaching-oriented setting—fixed camera angles, uniform indoor lighting, and a standardized pull-up bar—rather than generalizable reliability in unconstrained environments. Confusion matrix analysis further shows that the small 0.2% error rate occurs exclusively at the transient boundary between the Ascent and Arrival phases. Similarly, erroneous movement detection (e.g., leg bending) achieves 99.0% recognition under these controlled conditions, which fully meets the deterministic requirements of an educational demonstration.

### 4.5. Algorithm–Hardware Integration Analysis

PEPoseNet is used in this work as a lightweight deployable model rather than as a general-purpose pose-estimation algorithm. This manuscript is not positioned as an algorithm-design paper, but as an educational platform study that investigates how the STM32N6 chip, its NPU-supported deployment toolchain, and a lightweight executable vision model can be integrated into engineering education and competition-oriented training. Therefore, conducting comprehensive SOTA benchmarking, extensive ablation studies, or full sensitivity analysis falls outside the main scope of this paper. Instead, PEPoseNet is engineered as a customized educational payload to validate the hardware–software co-design workflow. Its architectural success is evaluated by a strict multi-objective equilibrium: retaining functional structural understanding (83.8% PCK@0.2) while compressing the model footprint to fit entirely within the 4.2 MB on-chip SRAM, enabling deterministic 32 FPS inference on the 600-GOPS NPU.

Specifically, the dual-branch Heatmap–Keypoint architecture is adopted to improve keypoint localization while maintaining a compact inference pipeline. The feature reuse mechanism is introduced to reduce redundant computation and improve feature utilization efficiency, which is particularly important for embedded deployment. In addition, staged training is used to stabilize optimization and improve convergence for the joint prediction of 13 human keypoints and 2 bar keypoints. These design choices are therefore closely related to the requirements of edge-side inference on STM32N6, where algorithmic effectiveness must be considered together with hardware executability.

From the deployment perspective, the proposed model is integrated into a complete embedded workflow of Sensor → AI Inference → Result Output on the STM32N6 platform with an on-chip 0.6 TOPS NPU. This demonstrates that the algorithm is not only validated at the model level, but also realized within an actual end-to-end embedded system. The pull-up recognition application further verifies that the proposed method can support practical intelligent interaction and automatic counting in a real edge-AI scenario.

From the algorithm evaluation perspective, the current results show that PEPoseNet achieves 83.8% PCK@0.2 on the task involving 13 human keypoints and 2 bar keypoints, indicating that the lightweight model maintains effective pose understanding ability under embedded deployment constraints. A preliminary error inspection indicates that most localization errors occur in frames with rapid transitions between motion states, partial self-occlusion of the arms or shoulders, and blurred hand regions near the horizontal bar. In addition, because the teaching dataset was collected under relatively constrained indoor conditions, variations in camera angle, illumination, clothing texture, and body scale may affect keypoint confidence and downstream state recognition. These observations suggest that the current model is sufficient for the controlled educational pull-up scenario, but its stability in unconstrained environments still requires further systematic evaluation. To quantitatively evaluate the trade-off between accuracy and efficiency under edge constraints, we compared the unquantized FP32 model running on the Cortex-M55 CPU with the INT8-quantized model deployed on the Neural-ART NPU. The original FP32 model achieves a PCK@0.2 of 84.5%, but suffers from a high latency of 215.6 ms (4.6 FPS), which is unacceptable for real-time video processing tasks in competitions. By applying INT8 post-training quantization and leveraging the NPU, the inference latency drastically drops to 18.4 ms (yielding an 11.8× speedup). This massive efficiency improvement and memory footprint reduction come at the cost of only a marginal accuracy degradation of 0.7% (dropping to 83.8% PCK@0.2). This quantitative trade-off analysis clearly demonstrates that the minor loss in absolute precision is a highly favorable and necessary compromise to fulfill the stringent real-time, low-power constraints of embedded edge AI deployments.

These results show that the proposed platform can support the deployment of a lightweight pose-estimation model in a real embedded application. The latency and power measurements mainly reflect the benefit of using the STM32N6 heterogeneous architecture and its NPU deployment toolchain. The PCK and motion-state recognition results indicate that the selected model is sufficient for the controlled pull-up assessment task used in teaching. Broader claims about pose-estimation accuracy or general robustness are not made in this study.

## 5. Discussion

### 5.1. Comparison with Representative Mainstream Platforms

To better position the proposed platform, we provide a scenario-based comparison with two representative mainstream alternatives, namely ESP32-S3 as a conventional MCU-oriented development platform and Jetson Nano as a general-purpose embedded AI platform. This comparison is primarily qualitative and is intended to clarify differences in architectural positioning, deployment complexity, and educational suitability, rather than to provide a comprehensive quantitative ranking across all platforms. The specific comparison of STM32N6, Jetson Nano, and ESP32-S3 is outlined in Table 5.

Table 5 shows that the proposed platform occupies a different position from official STM32 evaluation boards and Linux-based AI kits. Official evaluation boards are mainly used to demonstrate device functions, while Jetson Nano or Raspberry Pi-based kits are more suitable for high-level AI prototyping. The proposed platform focuses on MCU-level embedded development with NPU-assisted inference. This allows students to work with interrupts, memory limits, power constraints, RTOS tasks, and model deployment within the same hardware environment.

ESP32-S3 is widely used in IoT and embedded teaching scenarios due to its low cost, low power consumption, and strong support for peripheral interfacing and control-oriented applications. However, as it does not provide a dedicated NPU, its on-device AI capability is mainly limited to highly lightweight TinyML-style workloads. For vision tasks with higher computational demand, such as multi-keypoint pose estimation or real-time action recognition, ESP32-S3 typically requires aggressive model simplification and reduced input resolution, which may constrain both inference speed and recognition performance. Therefore, while ESP32-S3 is suitable for foundational embedded training, it is less effective as a unified platform for full-stack edge-AI practice.

In contrast, Jetson Nano is designed as a general-purpose embedded AI platform with stronger floating-point computing capability and a more mature Linux-based AI software ecosystem. It is well-suited for rapid prototyping of lightweight vision models and generally offers higher throughput than MCU-class platforms in tasks such as object detection, image classification, and pose-related inference. However, this advantage is accompanied by higher system complexity, greater power demand, and a development workflow that is more oriented toward application-level AI deployment than low-level embedded-system training. As a result, although Jetson Nano is effective for AI experimentation, it does not directly address the need for integrated training in sensor interfacing, driver development, RTOS-based task scheduling, and resource-constrained deployment.

The proposed STM32N6-based platform sits between these two categories, filling a practically important middle ground. Its integrated 0.6 TOPS NPU delivers far stronger edge inference than traditional MCU platforms such as the ESP32-S3, while retaining the low-level embedded development experience essential for microcontroller education. Against the Jetson Nano, the STM32N6 does not aim to compete on raw AI throughput; instead, its strength is a more balanced mix of embedded control, real-time inference, compact deployment, and engineering training value. Notably, it supports a full Sensor → AI Inference → Result Output workflow, so students can build intelligent applications—from hardware access and driver debugging to on-device model deployment and feedback control—all on a single platform.

From the perspective of educational design, the distinction is even more significant. ESP32-S3 mainly supports basic embedded experiments, while Jetson Nano primarily facilitates AI application development under a high-level software stack. By comparison, the STM32N6 platform is coupled with a progressive practice framework spanning sensor and communication fundamentals, RTOS development, and edge-AI deployment, and is further aligned with competition-oriented assessment through project defense and code review. This makes it more suitable for cultivating full-stack engineering competence rather than isolated programming or model deployment skills.

Overall, the comparative analysis indicates that ESP32-S3 is advantageous for low-cost foundational MCU training, Jetson Nano is stronger in general-purpose AI prototyping, and STM32N6 provides a practical balance for the competition-oriented embedded edge-AI education scenario considered in this study. Its unique value lies not in outperforming all mainstream platforms in absolute computing power, but in bridging the gap between traditional embedded teaching and deployable intelligent systems through an integrated, resource-aware, and pedagogically structured platform.

### 5.2. Scientific Novelty of the Engineering Implementation

Although the platform is built from commercially available components and existing tools, including STM32Cube.AI, TensorFlow Lite Micro, and RTOS-based scheduling, the main work lies in their integration for teaching use. The board design, power protection, vision interfaces, expansion connector, model-deployment workflow, and project-based curriculum were developed together rather than treated as separate modules. The experimental results show that this integrated design can support real-time NPU inference, stable low-power operation, and repeated student use in laboratory and competition training.

From this perspective, the engineering novelty is reflected in three validated aspects. Firstly, the heterogeneous computing resources are partitioned into a deterministic control domain and an accelerated inference domain, enabling real-time vision inference without sacrificing MCU-level control behavior. Secondly, the power and interface subsystems are redesigned for repeated student use, where incorrect wiring, hot-plugging, and mixed power inputs are common and must be tolerated. Thirdly, the hardware design is coupled with a complete educational workflow, including dataset construction, model compression, INT8 quantization, NPU deployment, and project-based assessment. The measured latency, power consumption, long-duration stability, and user satisfaction data provide empirical evidence that the proposed system-level design achieves outcomes that cannot be obtained by simply using a generic development board without such educational integration.

### 5.3. Scope of Application-Level Validation and Workload Coverage

A limitation of the present study is that the application-level validation is mainly based on the PEPoseNet-based pull-up recognition system. Although this case is suitable for demonstrating the complete embedded edge-AI workflow from visual sensing and preprocessing to NPU inference and feedback output, it does not cover the full diversity of edge-AI workloads. In particular, this study does not experimentally evaluate image classification, object detection, speech recognition, anomaly detection, sensor-based time-series inference, or multimodal AI applications.

Therefore, the current results should not be interpreted as proving the universal applicability of the platform to all embedded AI scenarios. Instead, they demonstrate that the STM32N6 chip and its associated hardware–software ecosystem can be effectively integrated into a curriculum-oriented teaching platform and can support at least one representative, real-time, vision-based educational application under MCU-class memory, latency, and power constraints. This distinction is important because the primary objective of the present manuscript is educational platform construction and representative deployment verification, rather than exhaustive workload benchmarking.

In future work, we will extend the validation protocol to a broader set of AI workloads. Planned tasks include image classification using compact CNNs, object detection using lightweight YOLO-style models, keyword spotting and speech-command recognition using audio front-ends, vibration- or current-based anomaly detection for sensor signals, and time-series classification based on IMU or environmental sensor data. These experiments will allow a more comprehensive assessment of the platform’s generality, toolchain compatibility, memory scalability, and pedagogical adaptability across different embedded edge-AI scenarios.

### 5.4. Evaluation of Pedagogical Effectiveness and Practical Outcomes

The segment presents the application status and achievements of the innovative teaching practice platform for embedded edge AI computing.

The proposed STM32N6-based teaching platform was initially developed in 2023. After several version updates, it was officially put into use in 2024. Currently, it has been fully applied in the pre-competition training and teaching for the National College Student Embedded Chip and System Design Competition, the National College Student Electronic Design Competition, and other innovation activities.

#### 5.4.1. Pre-Course and Post-Course Learning Assessment

To quantitatively evaluate students’ actual learning outcomes, a pre- and post-course assessment was conducted with 52 undergraduate students who participated in the STM32N6-based embedded edge-AI training program in 2024. The assessment consisted of five modules: embedded peripheral configuration, sensor communication protocols, RTOS task scheduling, edge-AI model quantization, and STM32Cube.AI-based deployment. The same knowledge framework and comparable question difficulty were maintained in the pre- and post-course tests. Each test was scored on a 100-point scale.

As shown in Table 6, the average total score increased from 52.4 ± 11.6 before the course to 82.7 ± 8.9 after the course, corresponding to an absolute gain of 30.3 points. A paired-sample *t*-test indicated that the improvement was statistically significant (*p* < 0.001). Among the five modules, the largest improvements were observed in edge-AI model quantization and STM32Cube.AI deployment, indicating that the platform effectively supported the transition from conventional embedded programming to deployable edge-AI engineering practice.

**Figure 8:**
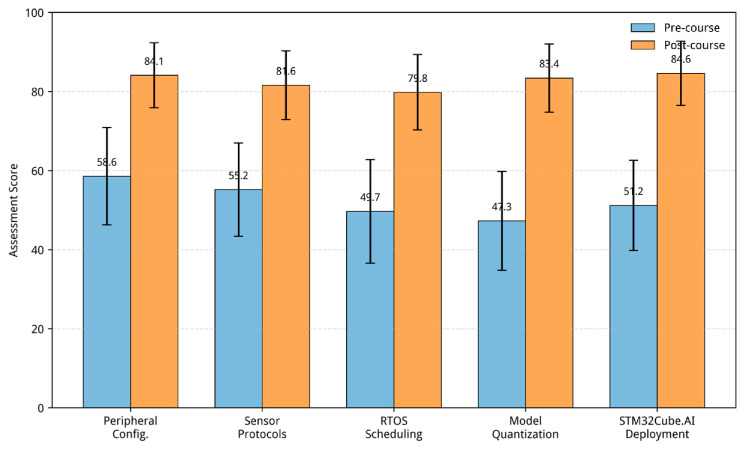
Comparison of pre-course and post-course knowledge assessment scores across five embedded edge-AI learning modules.

Figure 8 further visualizes the pre- and post-course score differences across the five learning modules. The results show consistent improvement in all modules, with particularly large gains in AI-related topics such as model quantization and STM32Cube.AI deployment. This suggests that the proposed platform not only supports conventional embedded-system instruction but also strengthens students’ understanding of the complete edge-AI deployment workflow.

#### 5.4.2. Practical Skill Improvement and Comparison with Traditional Teaching

In addition to knowledge assessment, students’ practical engineering skills were evaluated using a rubric-based scoring method. The rubric assessed five dimensions: peripheral driver development, sensor integration, RTOS-based task organization, AI model deployment, and system-level debugging. Each dimension was scored on a five-point scale by instructors according to students’ project demonstrations, code reviews, and debugging records.

The results are summarized in Table 7. The overall practical skill score increased from 2.14 ± 0.63 before the training to 4.02 ± 0.48 after the training. The most significant improvement was observed in AI model deployment, which increased from 1.72 ± 0.58 to 4.01 ± 0.51. This result indicates that the STM32N6-based platform helped students move from isolated embedded experiments toward complete edge-AI system implementation.

#### 5.4.3. Practical Outcomes

**Figure 9:**
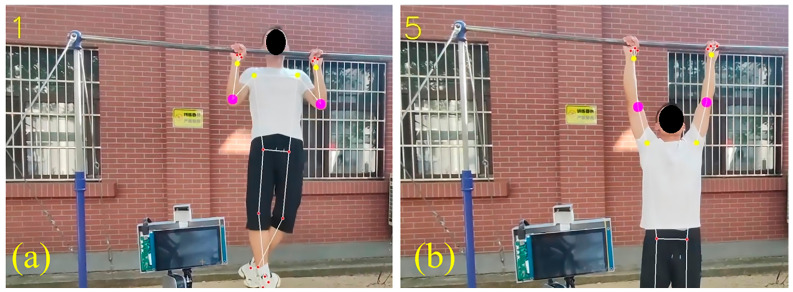
Practical Application Case of the Proposed Platform: (**a**) Counting 1; (**b**) Counting 5.

The platform has been used in pre-competition training and project development since 2024. More than 50 students have used the board in laboratory exercises and competition-oriented projects, accumulating over 600 h of practical use. Figure 9 shows one teaching application based on pull-up recognition. Several student teams using the platform completed embedded edge-AI projects and obtained awards in competitions such as the National College Student Embedded Chip and System Design Competition and the National Postgraduate Robot Innovation Competition. These outcomes suggest that the platform is useful for project-based training, although they should be interpreted together with the controlled experiments and learning-assessment results rather than as independent proof of technical superiority.

In recent practical applications, students trained on this platform successfully deployed edge AI technologies in complex scenarios, securing honors such as the National Second Prize in the 7th Embedded Competition and the 6th National Postgraduate Robot Innovation Competition. Within the scope of engineering education research, these standardized, blind-review competition outcomes serve as quantitative indicators. These outcomes demonstrate that the platform not only performs robustly in controlled settings but also translates technical advantages into pedagogical benefits, fully aligning with modern technology competition teaching concepts that emphasize the deep integration of edge AI into embedded projects [29]. Specifically, implementing a hierarchical training content system centered on competition projects effectively enhances students’ proficiency in applying these edge AI technologies [30]. By combining this hierarchical approach with targeted teaching cases and project templates, the platform significantly flattens the learning curve. It enables students to bypass low-level hardware debugging and focus on algorithm deployment and system integration, effectively shortening the project development cycle in highly constrained competition environments [31].

### 5.5. User Satisfaction Survey and Analysis

To quantitatively evaluate the platform’s practical pedagogical value, a user satisfaction survey was conducted among 52 undergraduate students who utilized the STM32N6 platform for pre-competition training and project development during the 2024 academic year. The questionnaire was specifically designed to align with the platform’s key architectural features, utilizing a 5-point Likert scale (1 = Strongly Disagree to 5 = Strongly Agree). The survey dimensions and aggregated results (Mean ± Standard Deviation) are detailed in Table 8.

As shown in Table 8, the overall student feedback is highly positive, with all items scoring above 4.6. The highest rated aspect was the hardware protection design (Q1, 4.85), corroborating that the reverse-polarity and overcurrent protection circuits are crucial for novice developers in open-ended competition environments. Furthermore, Q6 (4.82) and Q3 (4.80) highlight the success of the software-hardware co-design: the STM32Cube.AI toolchain integration effectively mitigated the steep learning curve typically associated with heterogeneous NPU deployment.

In the open-ended feedback section, several students noted that the MCU/RTOS-based control workflow of the Cortex-M55, combined with parallel NPU acceleration, provided a clearer structure than Linux-based MPUs when designing real-time robotic control projects. These survey results indicate that students generally perceived the platform as stable, usable, and helpful for embedded edge-AI project development. However, the survey should be interpreted as evidence of user acceptance and perceived learning support, rather than as a direct measurement of learning effectiveness. The actual learning gains and skill improvements are instead supported by the pre-/post-course assessment and rubric-based practical evaluation reported in Section 5.4.

### 5.6. Limitations and Future Work

The current validation mainly focuses on teaching use and a representative pull-up recognition case. Further work is needed to evaluate the physical limits of the board, its behavior under different AI workloads, and its applicability to broader embedded edge-AI teaching scenarios.

Validity and Broader Relevance. This study has several limitations regarding validity. In the first place, in terms of internal validity, learning outcomes were assessed using a pre/post design with the same student cohort. This approach captures improvement from using the platform but cannot fully isolate confounding factors such as prior experience, instructor guidance, training duration, or outdoor study. We mitigated this risk by keeping the knowledge framework, test difficulty, rubric-based project evaluation, and student cohort consistent.

Furthermore, for external validity, the evaluation involved 52 undergraduates from a single institution, and the platform was tested primarily through a pull-up recognition case and competition-style tasks. The results should therefore not be generalized to all universities, student populations, or embedded AI courses. Further studies across diverse institutions, student backgrounds, and AI tasks are needed to support broader conclusions.

In addition, regarding construct validity, the satisfaction survey mainly reflects perceived usability, stability, and learning support rather than direct measures of learning gain. While these offer stronger evidence of learning improvement, they do not capture the full scope of engineering competence, such as long-term retention, independent problem solving, or teamwork.

Lastly, although designed for formal engineering education, the platform may also serve non-academic settings including vocational training, robotics clubs and low-power edge-AI prototyping. In these contexts, its value stems from portability, low power consumption, protected interfaces, and a complete workflow. Nonetheless, it is not intended as a replacement for industrial-grade vision systems or high-performance GPU platforms.

To address these limitations, our future work will proceed along three core dimensions:

Hardware-Level Optimization and Physical Validation. Before wider deployment across universities, we will perform more thorough board-level and physical characterization. While the baseline STM32N6 specifications—processor architecture, embedded SRAM, accelerator capacity, and peripheral interfaces—are documented in the official STMicroelectronics datasheet [25], direct board-level measurements remain essential for validating the integrated teaching platform. Future work will therefore cover precise power ripple characterization, thermal profiling under extreme load, CPU and NPU utilization during heterogeneous execution, memory bandwidth across the camera-to-NPU data path, maximum throughput validation for MIPI-CSI, SPI, and Octo-SPI interfaces, and high-frequency signal integrity analysis of the four-layer PCB. These measurements will guide iterative improvements to the hardware design.

Comprehensive Multi-Workload Benchmarking: Currently, the platform’s application-level validation is pedagogical and mainly relies on PEPoseNet as a representative vision-based functional baseline. Although this task effectively demonstrates the complete workflow of camera acquisition, INT8 quantization, NPU inference, and result feedback, it cannot fully characterize the platform’s applicability to all embedded edge-AI workloads. In subsequent research, we will construct a multi-workload benchmark suite covering lightweight image classification, object detection, speech-command recognition, anomaly detection, sensor-based time-series classification, and multimodal edge-AI tasks. This future benchmark will be used to systematically evaluate the STM32N6 platform in terms of operator compatibility, memory utilization, latency, power consumption, and curriculum adaptability across different teaching scenarios. In addition, the current algorithmic evaluation remains limited because PEPoseNet is used primarily as a deployable teaching workload rather than as a standalone algorithmic contribution. More detailed error analysis, reliable evaluation under varying lighting and camera viewpoints, sensitivity analysis with respect to input resolution and quantization strategy, and ablation studies on the dual-branch structure will be conducted in future work.

Scalability of Model Complexity and Multi-Task Deployment: The current experimental validation mainly uses the lightweight PEPoseNet model as a representative educational workload. This choice is consistent with the primary design objective of the platform, namely to provide a portable, low-power, and interface-rich embedded edge-AI teaching system rather than a high-performance general-purpose AI accelerator. The STM32N6 platform is particularly suitable for demonstrating the complete workflow of sensor acquisition, INT8 quantization, NPU-assisted inference, RTOS scheduling, and result feedback under MCU-class constraints. Future work will therefore focus on defining the practical scalability boundary of the STM32N6 platform by evaluating larger lightweight CNNs, model partitioning strategies, external memory utilization, sequential or scheduled multi-model inference, and multi-sensor input scenarios.

### 5.7. Availability and Reproducibility

The proposed STM32N6-based teaching platform is not a commercial training kit at the current stage. It is provided as an educational training prototype and an open hardware reference design for embedded edge-AI teaching and student competition practice. The platform is intended to help instructors and students reproduce the complete workflow from hardware construction to model deployment.

To support reproducibility, the project materials have been organized and made available through a GitHub repository: https://github.com/Kyriewang0915/pull-up-recognition (accessed on 15 July 2026). The repository includes the main hardware design files, schematic diagrams, PCB layout files, bill of materials, embedded source code, STM32Cube.AI deployment examples, model conversion scripts, and basic user documentation. These materials allow users to rebuild the board, modify the interface design, reproduce the PEPoseNet deployment workflow, and adapt the platform to other teaching cases.

## 6. Conclusions

This paper presented a modular embedded edge-AI teaching platform based on the STM32N6 microcontroller. The platform integrates the Cortex-M55 core, Neural-ART NPU, protected multi-power-domain circuitry, vision-oriented interfaces, and modular expansion into a single board for teaching and competition practice. Using PEPoseNet as a representative workload, the prototype achieved an inference-only latency of 18.4 ± 0.3 ms and an energy efficiency of 28.31 FPS/Watt, while the complete camera-based application pipeline operated at 32.0 FPS under a power budget below 3 W. Teaching deployment results, including pre/post-course assessment, practical-skill evaluation, and user feedback, suggest that the platform can support students in learning peripheral configuration, RTOS scheduling, model quantization, and NPU-based inference. Future work will extend the evaluation to additional AI workloads and perform more detailed board-level physical characterization.

## Figures and Tables

**Table 1:** Practical Training Content Schedule.

Training Stage	Training Content	Hours	Objectives and Skills
Foundation & Introduction	STM32 Development Environment Setup (STM32CubeMX + Keil MDK) GPIO Control: LED/Button Experiments	2	Familiarize with STM32 Development Workflow, GPIO Configuration and Debugging
	Peripheral Basics: ADC Reading Analog Signals (Potentiometer/Photoresistor) UART Communication (Serial Data Printing)	2	Master ADC Acquisition and Serial Communication
Sensors & Communication	I2C/SPI Protocol Application (Temperature & Humidity Sensors, e.g., DHT22 or BME280) Multi-sensor Data Fusion Acquisition	2	Master I2C/SPI Driver Development and Multi-sensor Coordination
	Wireless Communication Modules (ESP8266/Bluetooth Module) Cloud Data Upload	2	Implement WiFi/Bluetooth Communication and Remote Data Transmission
Real-Time Operating System	FreeRTOS Porting and Multi-task Programming (Parallel Execution of Sensor Acquisition and Communication Tasks)	2	Master RTOS Task Scheduling and Resource Management
Edge AI Fundamentals	Edge AI Concepts and STM32 Performance Boundaries (CPU/Memory Constraints) Introduction to TensorFlow Lite Micro Framework	1	Understand Edge AI Characteristics and STM32 Compatibility
	Lightweight Model Training (Training Simple Classification Models on PC, e.g., KNN or Tiny CNN)	2	Master Model Compression and Quantization Techniques
AI Model Deployment	STM32Cube.AI Toolchain Usage (Model Conversion and Optimization) Model Integration into STM32 Project and Inference	3	Model Deployment and Local Inference (e.g., Environmental Anomaly Detection)
Comprehensive Application	System Integration: Sensor → AI Inference → Result Output (LED/UART/Wireless) Performance Optimization and Debugging	2	Complete End-to-End System Development and Performance Tuning
Project Assessment	Group Defense and Results Presentation Code Review and Optimization Suggestions	2	Summarize Learning Outcomes and Cultivate Engineering Thinking

**Table 2:** Difficulty levels, recommended learning trajectories, and stage completion criteria.

Training Stage	Difficulty Level	Target User	Successful Completion Criteria
Foundation & Introduction	Beginner	All students	Correctly configure STM32CubeMX project, implement LED/Button control, read ADC values, and transmit data via UART with verified serial output
Sensors & Communication	Beginner	All students	Successfully read temperature/humidity sensor data via I2C or SPI, implement multi-sensor data acquisition, and complete at least one wireless data transmission task
Real-Time Operating System	Intermediate	Advanced students, Competition preparation	Port FreeRTOS to the platform, create at least three concurrent tasks with correct inter-task communication, and demonstrate stable multi-task scheduling
Edge AI Fundamentals	Intermediate	Advanced students, Competition preparation	Train a simple classification model, apply post-training quantization and explain the trade-off between model size and accuracy
AI Model Deployment	Advanced	Competition preparation	Convert a trained model using STM32Cube.AI, integrate the generated C code into an STM32 project, and achieve successful on-device inference with measurable latency
Comprehensive Application	Advanced	Competition preparation	Build a complete end-to-end system (Sensor → AI Inference → Result Output), optimize performance to meet real-time constraints, and document the integration process
Project Assessment	Advanced	Competition preparation	Deliver a group defense presentation, submit a reviewed code repository, and demonstrate the working system in a live evaluation session

**Table 3:** Quantitative Performance Benchmarks of the STM32N6 Platform under Controlled Load Conditions (25 °C).

Load Condition/Deployment Strategy	Latency (ms)	Throughput (FPS)	RAM Utilization (KB)	Flash Utilization (KB)	Peak Dynamic Power (W)
System Idle (Baseline)	N/A *	N/A *	128	256	0.45
CPU-Only (PEPoseNet–FP32)	215.6	4.6	685	1420	1.15
NPU Accelerated (MobileNetV2–INT8)	9.2	108.6	384	850	1.68
NPU Accelerated (PEPoseNet–INT8)	18.4	54.3	412	965	1.92
Full System (PEPoseNet–INT8 + Camera + LCD)	31.3	32.0	1536	965	2.62 †
Maximum Stress Test (CPU + NPU 100%)	N/A *	Max	840	1850	2.78

* N/A means Not Applicable. † The full-system mode value is an engineering upper-bound estimation. This value is derived from three key references: the measured power consumption of NPU-only PEPoseNet inference, the peak power recorded in the full CPU–NPU stress test, and the typical power overhead brought by peripheral operations including MIPI-CSI camera acquisition, RGB LCD backlight driving, DMA transmission, and data preprocessing and display feedback.

**Table 4:** Statistical Performance and Energy-efficiency Comparison Across Educational Edge-AI Platforms. Latency Statistics were Calculated from 10 Independent Cold-boot Sessions, with 1000 Inference Iterations per Session.

Platform	Architecture Focus	Latency Mean ± SD (ms)	95% CI of Latency (ms)	Effect Size vs. STM32N6	Peak Power (W)	Energy Efficiency (FPS/Watt)
STM32H743	Cortex-M7 (CPU Only)	268.4 ± 5.2	[264.7, 272.1]	>10	0.85	4.38
Raspberry Pi 4B	Quad Cortex-A72 (MPU)	45.6 ± 8.4	[39.6, 51.6]	4.58	5.20	4.22
STM32N6 (Proposed)	Cortex-M55 + NPU	18.4 ± 0.3	[18.2, 18.6]	Reference	1.92	28.31

**Table 5:** Comparison between the proposed platform and representative STM32 development boards and educational edge-AI kits.

Platform	Primary Design Goal	AI Acceleration	Real-Time Embedded Control	Educational Edge-AI Workflow	Student-Oriented Hardware Protection	Main Limitation
STM32F4/H7 teaching board	MCU programming & peripheral learning	No dedicated NPU	Strong	Classical embedded/tiny TinyML only	Usually limited	No real-time vision inference
Official STM32N6 eval board	Chip evaluation & ecosystem demo	Integrated NPU	Strong	Partial support, not curriculum-focused	Not student fault-tolerant optimized	For device evaluation, not competition teaching
ESP32-S3 educational kit	Low-cost IoT & basic TinyML education	No dedicated NPU	Moderate	Simple TinyML tasks	Moderate	Low throughput for pose estimation & high-res vision
RPi/Jetson Nano kit	High-level AI prototyping	CPU/GPU acceleration	Weaker (Linux scheduling)	Strong AI app development	Limited low-level wiring protection	High power, weak MCU-level relevance
Proposed STM32N6 platform	Full-stack embedded edge-AI education & competition	Neural-ART NPU	Real-time- oriented MCU/RTOS control	Full workflow: sensor → quantization → NPU → control	Dual-input, reverse-polarity, reverse-current, overcurrent, soft-start	Lower raw throughput vs. high-end GPU

**Table 6:** Pre-course and post-course knowledge assessment results (N = 52).

Assessment Module	Pre-Course Score	Post-Course Score	Absolute Gain
Embedded peripheral configuration	58.6 ± 12.3	84.1 ± 8.2	+25.5
Sensor communication protocols	55.2 ± 11.8	81.6 ± 8.7	+26.4
RTOS task scheduling	49.7 ± 13.1	79.8 ± 9.5	+30.1
Edge-AI model quantization	47.3 ± 12.5	83.4 ± 8.6	+36.1
STM32Cube.AI deployment	51.2 ± 11.4	84.6 ± 8.1	+33.4
Overall score	52.4 ± 11.6	82.7 ± 8.9	+30.3

**Table 7:** Rubric-based practical skill assessment before and after the STM32N6-based training program (N = 52).

Practical Skill Dimension	Pre-Training Score	Post-Training Score	Improvement
Peripheral driver development	2.61 ± 0.74	4.28 ± 0.51	+1.67
Sensor integration	2.34 ± 0.69	4.06 ± 0.54	+1.72
RTOS-based task organization	1.98 ± 0.61	3.82 ± 0.56	+1.84
AI model deployment	1.72 ± 0.58	4.01 ± 0.51	+2.29
System-level debugging	2.05 ± 0.65	3.94 ± 0.49	+1.89
Overall practical skill score	2.14 ± 0.63	4.02 ± 0.48	+1.88

**Table 8:** User Satisfaction Survey Questionnaire and Aggregated Results (N = 52).

Evaluation Dimension	Specific Questionnaire Items	Mean Score	Standard Deviation
Hardware Stability & Reliability	Q1: The multi-power domain and protection circuits effectively prevented hardware damage during frequent hot-swapping and wiring errors.	4.85	0.36
	Q2: The modular interfaces (MIPI-CSI, 80-pin connector) made it easy to connect external sensors and cameras without complex breadboard wiring.	4.72	0.45
Edge AI Toolchain Usability	Q3: The integration with STM32Cube.AI significantly simplified the process of quantizing and deploying the INT8 AI models to the device.	4.80	0.40
	Q4: The end-to-end data flow (Sensor → NPU Inference → Output) was clear and easy to implement based on the provided project templates.	4.65	0.52
Pedagogical Effectiveness	Q5: Compared to traditional MPUs (e.g., Jetson Nano), this platform helped me better understand the low-level trade-offs between hardware constraints and AI performance.	4.78	0.42
	Q6: The platform effectively lowered the learning curve, allowing me to focus on algorithm tuning rather than low-level hardware debugging.	4.82	0.38
Overall Applicability	Q7: Overall, the platform meets the deterministic real-time and power constraints required for embedded technology competitions.	4.75	0.46

## Data Availability

The original contributions presented in this study are included in the article. Further inquiries can be directed to the corresponding author.
